# Common Mental Disorders in Smart City Settings and Use of Multimodal Medical Sensor Fusion to Detect Them

**DOI:** 10.3390/diagnostics13061082

**Published:** 2023-03-13

**Authors:** Ahmed Alwakeel, Mohammed Alwakeel, Syed Rameem Zahra, Tausifa Jan Saleem, Mohammad Hijji, Sami S. Alwakeel, Abdullah M. Alwakeel, Sultan Alzorgi

**Affiliations:** 1 Faculty of Computers & Information Technology, University of Tabuk, Tabuk 71491, Saudi Arabia; 2 Department of Computer Science and Engineering, Netaji Subhas University of Technology, Delhi 110078, India; 3 Department of Electrical Engineering, Indian Institute of Technology, Delhi 110016, India; 4 Department of Computer Engineering, College of Computer and Information Sciences, King Saud University, Riyadh 11543, Saudi Arabia; 5 Faculty of Medicine, University of Tabuk, Tabuk 71491, Saudi Arabia

**Keywords:** smart city, data fusion, mental health, depression, smart health

## Abstract

Cities have undergone numerous permanent transformations at times of severe disruption. The Lisbon earthquake of 1755, for example, sparked the development of seismic construction rules. In 1848, when cholera spread through London, the first health law in the United Kingdom was passed. The Chicago fire of 1871 led to stricter building rules, which led to taller skyscrapers that were less likely to catch fire. Along similar lines, the COVID-19 epidemic may have a lasting effect, having pushed the global shift towards greener, more digital, and more inclusive cities. The pandemic highlighted the significance of smart/remote healthcare. Specifically, the elderly delayed seeking medical help for fear of contracting the infection. As a result, remote medical services were seen as a key way to keep healthcare services running smoothly. When it comes to both human and environmental health, cities play a critical role. By concentrating people and resources in a single location, the urban environment generates both health risks and opportunities to improve health. In this manuscript, we have identified the most common mental disorders and their prevalence rates in cities. We have also identified the factors that contribute to the development of mental health issues in urban spaces. Through careful analysis, we have found that multimodal feature fusion is the best method for measuring and analysing multiple signal types in real time. However, when utilizing multimodal signals, the most important issue is how we might combine them; this is an area of burgeoning research interest. To this end, we have highlighted ways to combine multimodal features for detecting and predicting mental issues such as anxiety, mood state recognition, suicidal tendencies, and substance abuse.

## 1. Introduction

Two important events occurred at the turn of the new millennium that are likely to have had far-reaching consequences for human society. Firstly, because of a dramatic increase in urbanization over the past two hundred years, most people now live in cities. Presently, more than half of the world’s population resides in urban areas, and that number is projected to climb to 70 percent by the year 2050 [1]. Second, while the disability and death burdens of most diseases have gone down over the past 30 years, the burden of mental disorders, such as depression, anxiety, and substance use disorders, has gone up. As such, it has become imperative for the planners to consider how mental health and wellness might be integrated into the physical infrastructure of a smart city.

People move to cities for a variety of reasons, but one of the most important ones is the chance to learn and grow. Smart cities offer convenience to individuals by automating everything around them, from baby monitors to high-end military devices. Even though there is still disagreement on the definition of the term “smart city”, the modern consensus regarding its meaning points to the following characteristics: smart health, smart mobility, smart energy, smart economy, smart homes, smart infrastructure, smart citizens, and smart governance. In its simplest terms, the “smart city” can be described as an approach to city planning that relies heavily on information and communication technology (ICT) to monitor and, as a result, integrate and optimize the conditions and usage of a city’s lifelines, such as roads, bridges, tunnels, railway lines, seaports, airports, electricity, water, communication, etc., and an approach that effectively plans their management.

As such, cities in general and smart cities in particular provide huge convenience and comfort to people, besides giving them opportunities for growth. However, the way in which smart cities are now growing and changing, it almost seems as though only a robotic mind could survive. Today, we know that city life is harmful to the physical well-being of individuals [1]. For example, cardiovascular and respiratory illnesses are on the rise, particularly in cities. However, it has only recently been acknowledged that city life might be detrimental to one’s mental health. The acquisition of depression is, by far and away, the leading cause of disability worldwide [2]. This contributes to a depressed mood as well as a sense of helplessness. According to a number of studies [3,4,5], the proportion of people living in urban areas who are affected by this issue is twenty percent greater than that of those living in rural areas. Another factor is the risk of developing psychosis, which is reported to be 77% greater among urban dwellers compared to those who live in rural areas. In a similar vein, the possibility of having a generalized anxiety disorder is also increased by 21% [6].

Suicide rates are of even greater concern. Approximately one person takes their own life every 40 s. Among the world’s youth, this is the second leading killer. Results demonstrate that the likelihood of developing a mental disorder as an adult increases the longer a person spends their formative years in urban settings. There is a growing societal and economic cost associated with common mental disorders (CMDs) because of the increased risk of morbidity and mortality they bring. The COVID-19 epidemic is also having a significant and ongoing impact on people’s mental health around the world [7]. Living in cramped quarters and dealing with heavy crowding in public spaces are two examples of the unique stresses faced by city dwellers. Together, these factors strengthen the case that metropolitan environments contribute to the development of mental illness.

Smart healthcare is a platform for clinical systems that integrates technologies such as wearable devices, the Internet of Things (IoT), and wireless communication to facilitate the navigation of health information, the linking of individuals, resources, and organizations, and the intelligent handling of and response to the demands of the health environment. Disease prevention, diagnosis, treatment, follow-up care, healthcare management, individual patient autonomy, and medical discovery are some of the many components that make up this intricate system of smart healthcare. Its foundations are digital systems like the IoT and sensors, high-speed internet, edge and cloud networking, big data, next-generation wireless communication, machine learning (ML), and artificial intelligence (AI), as well as emerging biotechnologies.

As computer technology, digital signal processing, and automation improve, sensors are being used in more and more spheres of our lives. The information collected by sensors can help doctors identify dangerous situations quickly and accurately, and help patients to be more aware of their symptoms and changes. From measuring body temperature to controlling dialysis, invasive and non-invasive technologies provide personalized data and digital information to help patients and the healthcare industry [8,9,10]. Similarly, the IoT has become part of both the practitioner and patient sides of healthcare. For example, ultrasound, blood pressure, glucose sensors, EEG, and ECG devices are starting to talk to each other so that patients can better control their health, especially when they need to attend multiple follow-ups. Smart wristwatches, Fitbits, activity trackers, augmented reality glasses, and smartphones are some of the examples of smart healthcare IoT devices, popularly called the Internet of Medical Things (IoMT) [11,12]. Memory, judgment, understanding, a sense of direction, etc., are all part of mental functions (MF). Medical implants and convenience implants are used to keep track of these functions. For example, the Apple Watch and the Kardia Band can detect atrial defibrillation, which is a cause of strokes.

Mental diseases are complicated, and often cause symptoms that overlap and affect different parts of our bodies. It can be hard, then, if not impossible, to keep an accurate eye on a patient using just one medical signal modality or IoT device. Gross motor activity, body movements, speech, and motor reaction time are all areas in which people with mental health problems are different from normal and psychiatric comparison groups. These psychomotor symptoms are easier to identify than electroencephalogram (EEG) signals, and yet they have a high discriminative validity for the purpose of diagnosing mental illnesses. It is normal practice for professionals to record these behavioural signals in a variety of modalities, such as speech, body language, and written words, during clinical interviews before arriving at a final diagnosis [13]. Even though one modality almost never gives all the needed information, the complementary nature of the symptoms means that each modality provides new or different information that none of the other modalities do. Because of this, multimodal systems are needed, and medical signal fusion innovations are required.

The distinguishing features of this work are as follows.

- It highlights the major factors that lead to growth of mental disorders in cities, and proposes countermeasures to deal with them.
- Most state-of-the-art methods focus on a single mode for feature extraction, which leads to performance degradation. By proposing the use of multimodal feature fusion for the detection of mental illnesses, this work provides an additional tool for identifying mentally disturbed individuals.
- It sketches out the major symptoms and signs of mental issues prevalent among cities that can be identified through multimodal sensor fusion.
- It proposes the use of a multimodal fusion framework to generate accurate estimates of depression, anxiety issues, substance abuse and suicidal tendencies.

The remainder of the paper is structured as follows. Section 2 identifies common mental disorders, and their signs and symptoms. To extrapolate the significance of our work, this section highlights the prevalence of these disorders in cities. Section 3 focuses on identifying the factors that contribute to the growth of mental disorders in cities. In Section 4, we identify multimodal feature fusion as an option for detecting and predicting mental disorders in real time. Here, we also present a critical analysis of the most recent and relevant state of the art method, discussing both its pros and cons in diagnosing mental illnesses. Finally, Section 5 concludes the paper.

## 2. Common Mental Disorders

Signs and symptoms are different, but they describe the same condition. Signs are things that can be seen by a doctor or someone else who is not the patient. Symptoms are what the patients think they have. People can say that a disorder’s symptoms are what make it what it is, and that the disorder’s signs show that it is there.

### 2.1. Major Depressive Disorder (MDD)

**Figure 1:**
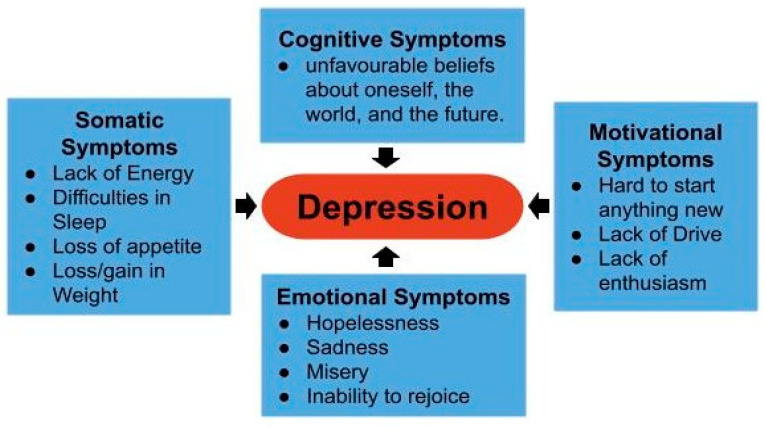
Various facets of MDD [14,15,16,17,18].

If a person has never experienced mania, hypomania, or a mixed episode, but has suffered from a major depressive episode, they are considered to have a major depressive disorder. In this case, the individual is markedly depressed or disinterested in previously enjoyable activities for the vast majority of each and every day. Figure 1 presents the various facets of MDD.

**Signs and Symptoms:** Signs of MDD may include irritation or aggression, slower movement or speech, restlessness or difficulty sitting still, oversleeping or an early morning wake up, and weight fluctuations.

Among the symptoms of MDD are the following [14]:

- Low mood: This is qualitatively distinct from regular moments of sadness or grief that everyone experiences. Some suffer from sobbing bouts or the desire to cry, whereas others experience a lack of emotional reaction [15].
- Loss of Interest/Pleasure: MDD is also characterised by a loss of interest in formerly rewarding activities. Sorrow may also manifest as apathy or boredom. The loss of sexual interest, desire, or function is also common [14].
- Sleep problems: The most common symptom is waking up early in the morning and not being able to go back to sleep (terminal insomnia). Early insomnia describes when people find it hard to fall asleep at the start of the night. Anxiety is often present at the same time [16].
- Energy: People with depression often say they are tired, have low energy, or both. They also have trouble getting started or starting tasks. This tiredness can be physical or mental, and it may be caused by not getting enough sleep or food [15].
- Guilt: People who are depressed may misunderstand small, everyday things and blame themselves for bad things that are out of their control, which can sometimes be a sign of delusion [17].
- Concentration: People who are depressed often have trouble paying attention and making decisions. Most complaints about memory are caused by trouble paying attention and getting side-tracked [17].
- Weight: Reductions in appetite, taste, and enjoyment of food can result in a considerable loss in body weight. However, some individuals may turn to “comfort” foods such as sugary snacks and starchy carbs when they are feeling down. When people overeat and stop exercising, they gain weight, which can have a negative effect on their sense of self-worth [14].
- Movement latency: Psychomotor alterations include the slowing of body motions, loss of facial expression, and silence. Anxiety can also manifest as agitation in the body, leading to rapid speech, restlessness, and other symptoms of psychomotor agitation [16].
- Suicidal tendencies: Nearly two-thirds of depressed persons have suicidal thoughts of some kind, ranging from momentary wishes that life would end to detailed suicide plots [18].

**Prevalence:** An estimated 3.8% of the world’s population suffers from depression. This number rises to 5.0% among adults and 5.7% among individuals aged 60 and older [19,20]. About 280 million people worldwide suffer from depression. Table 1 identifies the five most depressed countries in the world as of 2022, adjusted by the World Health Organization (WHO) as per population size.

### 2.2. Anxiety Disorder (AD)

Anxiety is a broad feeling of concern about potential future danger, catastrophe, or misfortune [22]. Muscles may stiffen up, breathing may quicken, and pulse rate may increase as a result of anxiety, all in an effort to protect the body from the perceived threat. Although commonly used interchangeably, anxiety and fear are theoretically and physiologically distinct states. Fear is an alarm reaction that happens in response to an imminent danger, i.e., fear is a proper, present-focused, and brief response to a clearly visible and precise threat, whereas anxiety is a future-oriented, long-acting response that is largely focused on a diffuse threat.

**Signs and Symptoms:** In the past, the most common way to tell the difference between fear and anxiety responses was to see if there was a clear and obvious source of danger that most people would agree was real. When the danger is clear (for example, “I am afraid of snakes”), the feeling is called fear. When we have anxiety, on the other hand, we often cannot say what the danger is (e.g., “I am worried about my parents’ health”).

Fears and worries that have no basis but cause severe suffering and/or functional impairments are characteristic of anxiety disorders. It is also worth noting that many people who suffer from one type of anxiety disorder will also suffer from another type of anxiety disorder and/or depression at some point in their lives, either simultaneously or later [23,24]. Considering these shared features, it’s not surprising that the root causes of anxiety disorders share key characteristics (as well as many differences). The Diagnostic and Statistical Manual of Mental Disorders (DSM) includes the following disorders in its list:

- Specific phobias: Those who suffer from specific or social phobias react with anxiety or panic not only when confronted with the thing or scenario they fear, but also at the mere thought of coming into contact with whatever it is that causes them distress. People may even know that they are overreacting (for example, extreme fear of birds, spiders, dogs, etc.) but it does not stop them from being paralysed by it [24,25].
- Social phobias: A person with social anxiety disorder worries a lot about being embarrassed, humiliated, rejected, or looked down on in social situations. This worries them a lot and makes them feel bad. They try to avoid these things or deal with them with a lot of worry. People may be very afraid to speak in public, meet new people, or eat or drink in public, for example [26].
- Panic disorder: People who suffer from panic disorder are prone to having frequent panic attacks, in addition to extreme anxiety that is centred on the prospect of having another one. Many of these signs and symptoms cluster together during an attack: heart palpitations, pounding, or a racing heart; symptoms such as perspiring, shivering, or shaking; experiencing a sensation of being suffocated; pain in the chest, feeling weak, a sensation of suffocation, numbness or tingling, shivering or hot flushes, discomfort in the stomach or feeling sick; feeling negatively emotionally invested, the terror of helplessness and the terror of death. Due of the severity of the symptoms, many persons experiencing a panic attack incorrectly assume they are having a heart attack or some other potentially fatal condition [27].
- Agoraphobia: Individuals who suffer from agoraphobia will go to tremendous measures to avoid a variety of dreaded scenarios. Those who suffer from agoraphobia have irrational fears in at least two of the following situations: riding public transit, being in open areas, being in enclosed rooms, waiting in lines or crowds, going outside the house alone, etc. People with agoraphobia avoid them with great effort, always need to be with someone else, and suffer from crippling anxiety whenever they are forced to confront one of these situations alone. If left untreated, agoraphobia can become so severe that the sufferer feels trapped within themself [28].
- Generalised anxiety disorder (GAD): People with GAD tend to feel anxious and worried all the time, without any particular triggers or causes; they may have panic attacks occasionally, but they are not the main source of their distress. This constant worry and stress can cause physical symptoms such as restlessness, trouble focusing, tight muscles, or trouble sleeping. Often, the anxiety concerns things that happen every day, such as work, family, health, or small things such as chores, getting the car fixed, or going to an appointment [29].

Prevalence: Approximately one-third of the general population, or 33.7%, may suffer from an anxiety disorder at some point in their lives, according to large-scale surveys [23]. The lifetime frequency of certain phobias is estimated to be between 3 and 15 percent worldwide, with apprehensions and phobias of heights and animals being the most frequent [30]. Prevalence rates are shown in Table 2 for the three major community surveys [31,32,33]. These polls indicate that specific and social phobias are the two most prevalent anxiety disorders.

Moreover, the incidence of anxiety disorders in women is nearly twice as high as the prevalence in men, according to research that has been conducted repeatedly.

### 2.3. Substance Use Disorder (SUD)

The term “substance” refers to any chemical that, when swallowed or otherwise taken into the body, can have negative effects. Abuse of substances involves both habitual use of illegal substances and the inappropriate use of prescription medications. Cannabis, ecstasy, charas, bhang, opium, alcohol, cigarettes, and psychoactive substances are all quite popular. Heroin, however, is by far the most widely used illicit substance worldwide. When it comes to substance usage, young people are especially vulnerable.

**Signs and Symptoms:** Substance misuse among adolescents is connected with a decline in academic performance, greater absenteeism from school and extracurricular activities, and an increased risk of dropping out. Some other important signs and symptoms include:

- Intoxication-related injuries: a disproportionately high number of young people who use alcohol and other substances are at increased risk of dying from suicide, violence, accidents, and diseases. Worldwide alcohol consumption was a contributing factor in the deaths of over 2 million individuals in 2016 [34]. Sexual contact between an infected person and another can result in the spread of HIV/AIDS. Teens who abuse substances are disproportionately represented among the population at risk for acquiring sexually transmitted diseases such as HIV/AIDS. Use of psychoactive chemicals (especially injectable ones) or actions prompted by impaired judgement and impulsivity while under the influence of such substances is included.
  Adolescent substance misuse is often associated with depression, developmental delays, apathy, withdrawal, and other psychosocial dysfunctions. Young people’s marijuana usage is associated with impairments in short-term memory, learning, and psychomotor skills. Similarly, motivation and psychosexual/emotional growth could be impacted.
- Relationships: Relationships are impacted by substance abuse in a wide variety of ways. Users risk social isolation and stigmatisation. Less time is spent interacting with others, and some may even isolate themselves. Users’ lack of trust, respect, and honesty with their partners/parents/peers is particularly noteworthy, as is the obvious shame and guilt that comes along with it. In dire circumstances, this can even lead to maltreatment of loved ones [35].

**Prevalence:** The lives of millions of drug users around the world are a living hell, somewhere on the edge of despair and death. The number of drug users in India is steadily rising as the country is ensnared in the cycle of addiction. In India, there are at least one million documented heroin users, and possibly as many as five million more. Males outnumber females as drug abusers. Adolescent cigarette smoking rates in Kenya, for instance, are 43% for males and 37% for females. Table 3 provides the annual prevalence (2019) of cannabis, opioids, opiates, amphetamines, and ecstasy in various regions [36].

## 3. Factors Contributing to Mental Issues in Cities

An increasing number of people are leaving rural areas and moving to urban centres in search of higher quality jobs, medical care, educational opportunities, and other amenities [37]. We observed that there has been a dramatic increase in the percentage of the population with CMDs in recent years. According to an estimate, about 46 million U.S. citizens aged 18 and up will have a major depressive episode by 2050. The increase will be more evident in those aged 65 or older [38]. This estimate may be reduced if measures are taken to reduce the prevalence, severity, and duration of depression. Internet of Things-based health monitoring systems (HMS) have been rapidly created to offer a workable alternative to conventional healthcare management strategies. The goal of HMS is to let people receive low-cost medical treatment from a distance, allowing them to keep their freedom while avoiding the hassle and rising costs associated with visiting traditional medical facilities. The United Nations Population Fund (UNFPA) estimates that by 2050, more than 2 billion individuals around the world will be aged 60 or older [39]. It has also been estimated by the World Health Organization (WHO) that by the year 2035, a shortage of 12.2 million medical professionals will exist throughout the world [40].

While we are beginning to grasp how city life affects physical health on a global scale, only a small number of experts are trying to understand the relationship between mental health and poorly constructed cities. There are not enough personnel or facilities to handle the delicate care required for dealing with people suffering from mental illnesses.

Most of the mental disorders discussed in this manuscript are primarily caused by loneliness, isolation, and stress. However, from where do they originate? According to the findings of epidemiological studies, the potential concerns reside inside the concrete landscape of cities. Some examples include:

- **Social inequalities:** Living in a city is a complicated affair which comprises opposing aspects that are often hard to explain. For example, there is a significant gap between a wealthy “garden city” resident and a poor suburban resident. In this case, numerous factors become apparent. The former has access to both green space and opportunity. In contrast, the latter lacks these facilities. In addition, there is a scarcity of quality homes.
  People who come to the city with pre-existing risk factors, such as low income, membership of a minority group, or mental health issues, frequently run into negative discrepancies. For instance, this can involve the physical and mental segregation of individuals into neighbourhoods that may be characterized by poverty and social challenges. This can give rise to feelings of injustice and hopelessness, as well as experiences of prejudice and discrimination, which may have an impact on mental health.
- **Decrease in social support:** This happens as the number of nuclear families increases. The rise of nuclear families in urban life has contributed to an overall increase in the number of incidences of violence committed against women. There is a correlation between alcohol misuse and the mental health of women, as well as intimate partner violence [41].
- **Development of fringe populations:** Because cities offer employment opportunities, many workers migrate to cities and make a living by earning on a daily basis. This is called a “fringe population” [42]. The connection between financial hardship and one’s mental health is intricate and multifaceted.
- **Cultural transition:** It is widely believed that one of the causes of psychological disturbance is the paradigm of cultural revolution, in particular the transition from rural to contemporary civilization. However, it is impossible to deny that the tension created by the transition from rural culture to urban culture is one of the variables that contribute to stress-related issues. A one-of-a-kind dynamic emerges when cultural variables are brought into play with urban dynamics. When it comes to the effective management of mental disorders in cities, having a good understanding of how cultural dynamics interact with adaptation to urban living may be helpful.
- **Environmental factors:** People can be affected in two primary ways by their urban environment: an increase in the number of stimuli and a reduction in the number of protective elements.
  *Overload:* People who live in cities are subjected to a heightened sensory level, which includes density, congestion, noise, odours, sights, disorder, pollution, and the intensity of other inputs. This can lead to feelings of overload. The entire metropolitan environment has been thoughtfully crafted to send specific meanings and signals to those who inhabit it. These impulses cause behaviour and thinking on a subliminal level of consciousness, and these become more powerful as the inability to “cope” with the situation becomes more apparent. This can have the effect of overload, which raises the body’s baseline levels of arousal, stress, and readiness while also driving people to seek relief in places that are quiet and private. Over time, this urge may develop into social isolation, which is associated with depression and anxiety [43].
- **Waning of protective factors**: People who live in cities may discover that they have less access to the factors that are protective for good mental health than those who live in rural areas. This may be the case due to the erosion of protective factors. For instance, individuals may have a limited access to nature, fewer opportunities to incorporate exercise as a part of their daily routines, and reduced leisure time as a result of the increasing amount of time spent working and commuting around the city.
  As a result of variables such as crowding, light, noise, and stress, individuals may experience feelings of insecurity and even less restful sleep. They may also have less privacy. When people move from rural areas to metropolitan areas, they frequently leave behind their robust social networks consisting of friends and family. It takes time to create social capital that is similarly supportive in the city. This may particularly be the case due to the fact that people who live in cities may be reluctant to participate in social interactions in order to avoid being overstimulated, out of concerns for their safety, or because of the decreased likelihood of developing future relationships with each individual they meet. As these protective variables become less prevalent, individuals are exposed to a greater risk of acquiring mental health issues [43].
- **Cyberterrorism:** With the growth of smart cities, cyberterrorism comes as a plus one. Cyberterrorism, like regular terrorism, tries to achieve political, economic, religious, or ideological goals by hurting civilians physically or mentally. A study by [44], summed up in Table 4 below, depicts the impact of cyber terrorism on people’s mental health. The data demonstrate that the severity of the cyber attack is correlated with a corresponding increase in stress and anxiety.

Table 4 unmistakably demonstrates that the effect of cyber attacks on individuals’ psyches is virtually identical to that of traditional terrorist strikes. In addition, it demonstrates that in the aftermath of cyber attacks, the threat perception rises rapidly. In the world of IoT-enabled smart cities, anxiety and fear levels among the populace are amplified, since individuals’ find their privacy at risk.

For this reason, progress in mental healthcare that takes intelligence into account is crucial. Smart health (s-health) is a new healthcare paradigm that emerged in response to the growing popularity of the smart city philosophy and the convergence of the concepts of electronic and mobile health. S-health is the delivery of health services by leveraging the context-aware network and sensing infrastructure of smart cities [45].

## 4. Multimodal Medical Sensor Fusion

Single-mode feature fusion and multimodal signal fusion are two types of medical signal fusion. Different medical signals have various features that describe different parts of human physiology. For example, the low-frequency and high-frequency parts of a heart rate time series tell us about how the parasympathetic and sympathetic nervous systems work. As such, combining them could give better results than using a single feature. Not only can different parts of a person’s physiology be taken into account with multimodal systems, but they also allow for missing data imputation, quality-aware fusion [46], and a better sense of experience. With the improvement of AI and ML algorithms (such as deep neural networks), high computing processing power, and large amounts of data storage in end devices, along with wireless communication technologies such as 5G and beyond, smart healthcare systems are growing to meet the needs of patients and stakeholders. This has made it possible to measure and analyse multiple signal types in real time and give clients and medical professionals feedback about how well diseases can be found and predicted. Multimodal medical sensor signal fusion will play a very important part in getting to this point.

Earlier studies have proven the efficacy of deep architectures on multimodal data [47,48,49]. Ref. [47] introduced bimodal deep autoencoders to capture the cross-modal correlations necessary for audio-visual speech recognition. Using multimodal deep belief network (DBN) models, Kim et al. [48] and Ranganathan et al. [49] reported improved classification performance over unimodal baselines when applied to emotion recognition. This was accomplished by fusing first-order representations from different modalities into a single shared hidden layer. However, the recognition of mental disorders is distinct from the recognition of emotions in that it requires the capture of the dynamic features of those feelings, as well as the temporal information from a period of variable length.

The majority of earlier investigations employed data from a single modality as the research subject, and typically retrieved features while the subject was at rest [50,51,52,53]. The acquired features, however, are relatively small as the features are solely extracted in a unimodal manner. This in turn results in insufficient medical data and lowers the performance of the classification process as a whole.

**Figure 2:**
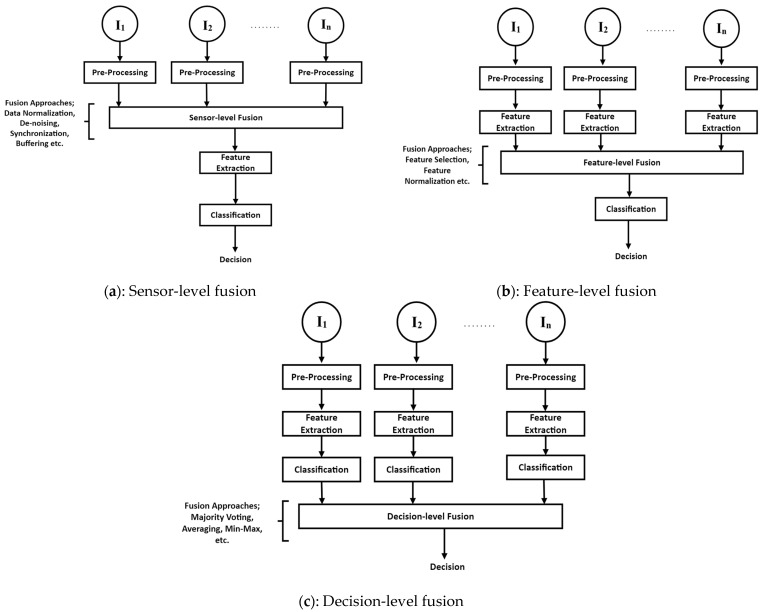
(**a**–**c**): Different types of data fusion [46,47,48,49,50,51].

Traditionally, there have been three ways to combine medical signals from different sensors (Figure 2);

- **Sensor-level fusion:** This is the process of combining data from multiple sensors into a single model for making decisions, i.e., different types of signals are pre-processed and then mixed. This method needs new ways of synchronizing sensors, buffering data, removing noise, and normalizing data [54]. It is a type of low-level information fusion in which multiple sensors collect and combine similar data about the same feature [55].
- **Feature-level fusion:** In the feature-level fusion, features from each sensor’s data are taken out separately and then put together. In this type of fusion, neural networks and probability statistics can be used to combine information from different sensors [55], i.e., features from multiple signals are extracted, fused, and finally classified using feature-level fusion. The issues seen in umimodal extraction of features are resolved by feature fusion because, in contrast to each modality’s individual feature, many modalities’ features can fully describe the medical data, resulting in feature complementarity. Early information fusion laid the groundwork for what later became known as “feature fusion” techniques. For research, data from several sensors were put together. In recent years, target tracking and recognition, pattern analysis, and classification have all greatly benefited from the use of data fusion [56,57]. This strategy calls for novel approaches to feature normalisation (for signals with varying amplitudes and time scales, for instance) and feature selection. After feature extraction is complete, feature-level fusion is performed by combining previously extracted features in either a linear or nonlinear fashion to produce new fusion features. The original information is not easily lost after fusion, and the method has strong real-time performance, both of which are important for the final categorization of results.
- **Decision-level fusion**: The highest level of fusion is at the decision level. It depends on signal processing and the classification of each modality, as well as a second classification step that combines the outputs (or decisions) of each classifier. The outputs of the classifiers can be combined in different ways, such as by majority vote, maximum likelihood, or confidence score. The quality ratings of each modality can also be used. In decision-level fusion, individual decisions are made in accordance with several feature sets, and then those individual decisions are coordinated or combined into a single global decision.

**Figure 3:**
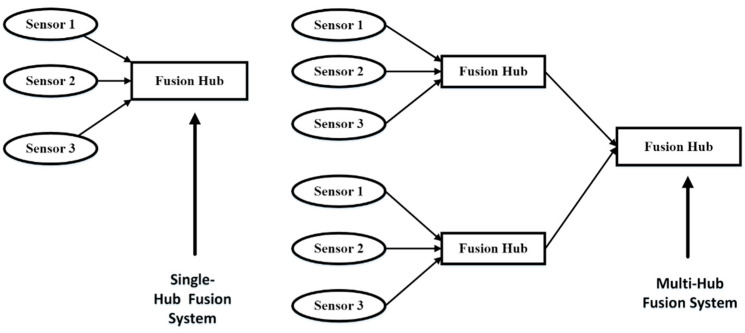
Levels of medical signal data fusion [46,47,48,49,50,51].

Additionally, data fusion can happen in a single hop or over several hops. Figure 3 shows the levels of medical signal data fusion.

The following sub-sections propose the utilization of multimodal medical sensor fusion for mental disorder detection in smart cities.

### 4.1. Anxiety and Stress Recognition Using Data Fusion in Smart Cities

The modern human lifestyle is extremely stressful and tense, leading to increased levels of anxiety and tension. Intensified negative feelings are a direct result of being exposed to stress. Anxiety and stress are linked to all of the above-cited mental issues, as well as depression, disruptive behaviour, emotional asymmetry, agitation, and a range of psychological problems, such as sensory dysfunction [58]. Positive emotions help with processing and perceptual learning, while negative emotions slow down working memory, which makes learning less effective.

IoT in smart cities enables monitoring of all important systems, yielding huge data streams, and can thus provide smarter decisions. Because of better communication, better technology, and sensors, among other things, healthcare services in smart cities can be completely customized to meet the needs of their citizens. Therefore, with IoT and automated learning methods, creating an intelligent framework can help to elucidate how stress affects emotions among people living in cities. Smart cities’ ability to facilitate monitoring is beneficial to health preservation. This method is non-invasive, and it can be used to evaluate how one handles common stresses. Accordingly, an individual’s pattern of sleep can be used as a proxy for estimating the amount of stress that they are under. Further, this is achieved by disseminating diagnostic data and smart home-related details to monitor and adjust stress levels in line with individual preferences and improve the quality of life. A number of studies in the literature have proposed models for anxiety and stress recognition. These include [59,60,61,62,63,64,65]. However, these approaches focus on a single physiological modality for anxiety and stress recognition, and hence are less effective. Medical signal fusion can determine stress levels by analysing physiological signals, such as electrocardiograms (ECG), electroencephalograms (EEG), temperature (TEMP), electrooculography (EOG), electromyograms (EMG), blood volume pressure (BVP), respiration (RESP), arterial blood pressure (ABP), electroglottography (EGG), mechanomyograms (MMG), magnetoencephalograms (MEG), photoplethysmograms (PPG), and galvanic skin responses (GSR), that come from different body sensors. It is possible, for instance, to use the ABP signal to predict tachycardia, because it contains data on hemodynamic changes and heartbeat rhythm. Negative emotions, anxiety, and stress all cause arousal in ECG and EEG signals. In order to better detect mental stress, information from EEG electrodes combined with data from functional near-infrared spectroscopy (fNIRS) can be used. For the purpose of emotion analysis, multiple EEG, EMG, and EOG channels can be fused [66,67]. With the help of EEG, EMG, and ECG fusions, breathing patterns can be tracked [68]. By fusing the data coming from all these sensors, a more accurate result about the stress and anxiety level of an individual can be obtained. Moreover, data fusion can be used for analysing video clips, measuring citizens’ emotions, and quantifying the intensity of arousal. An efficient prediction scheme can be developed for stress prediction through the use of a correct fusion model in conjunction with an innovative gunner algorithm (AIG).

### 4.2. Mood State Recognition Using Data Fusion in Smart Cities

A number of approaches have been proposed in the literature for mood state recognition. These include [69,70,71,72]. However, these mood state recognition approaches use a single modality as the input to the machine learning models and hence are less effective. The following depicts the importance of multimodal sensor fusion for mood state recognition. An EEG can be utilised as a reliable indicator of mood states. It has been found that the alpha wave may be related to a person’s level of relaxation [73]. Strong alpha waves are associated with a low brain behaviour index. Stable alpha behaviour suggests a high level of brain activity [74]. In contrast, the beta wave is associated with the thought state, which is prominent in the frontal cortex and surrounding regions. According to [75], the beta/alpha ratio may reflect a person’s mood and the presence of depression. A low beta/alpha ratio indicates a low frequency of negative emotions, whereas a high ratio indicates an active state. In addition, the comparison of alpha and beta activities between depressed patients and healthy subjects indicates that depressed patients have greater alpha and beta activities.

Similarly, analysis of high-frequency heart rate variability (HRV) is typically utilised in the practise of diagnosing major depression in patients. According to Pizzi et al. [76], depression and the HRV each have their own independent relevance, and together they may increase the likelihood of a patient having heart disease. Physically healthy patients with mood and anxiety disorders and alcohol dependence have been shown to have lower HRV, as discussed by the authors in [77,78]. As a result, depression risk increases with decreasing HRV. Major depressive disorder (MDD) patients are reported to have lower HRV by the authors of [79]. They hypothesised that a decrease in HRV represents a psychophysiological marker of MDD. Those with MDD and co-occurring GAD have the lowest HRV.

As such, the assessment of the mood state can be carried out in different ways, but if only one method is used, it may not be as reliable. So, a high-level information fusion technique is required to combine the signals from different devices into a single value. This can be carried out using Bayesian modelling, wherein emotion attributes such as irritability, lack of energy, lack of sleep, etc. are first translated into numerical values, and then different conditional probabilities are set based on an expert/doctor’s advice. At last, a Bayesian network [80] is used to obtain the probability values of depression/mood state.

### 4.3. Suicidal Tendency Detection Using Text and Graph Fusion

Many people who have committed suicide, especially in recent years, have turned to social media to communicate how they feel [81]. It has been found that people who are depressed spend a lot more time on the internet and social networking sites than people who are not depressed [82,83]. Additionally, depressed people who used social networking sites said they felt less happy, more worthless, guilty, distracted, irritable, and had suicidal thoughts a lot more often. Young people who are depressed talk about their feelings on social media networks. Previous deep learning approaches always relied on text to identify depressed users [83], giving scant attention to the fact that user-posted images can also convey emotion. Many posters use accompanying images that convey the intended mood of their posts in some way. Subsequently, to identify signs of suicidal tendencies among social media users, a fusion of graphical and textual cues could be used. Users’ states of mind could be described by analysing features such as his/her user profile, social network, domain, and topic-specific comments/reactions.

### 4.4. Recognizing Illegal Drug Dealers Using Multimodal Data Fusion

The distribution of illegal drugs represents a significant risk to cities in general, and to the younger generation in particular. Substance abuse has been linked to a wide range of negative societal aspects such as traffic mishaps, murders, infectious diseases, and deaths. With the development of social media into an open platform that can express any kind of social activity, drug use can now be encouraged on this platform, which can then lead to the abuse of drugs by other individuals. When people use drugs on social media, it also serves as a prominent platform for the distribution and sale of illegal drugs. On social media, drug dealers advertise their services by posting images of various types of drugs along with their contact information. When there are millions of people using the same social media platform, it can be very difficult for law enforcement agencies to trace drug dealers. Because of this, the automatic detection of drug dealers, along with the types of drugs they sell and their contact information, is extremely important. According to studies, a sizable number of drug-related transactions [84,85] take place on social media platforms.

Utilizing a feature-level fusion technique, it is possible to identify the type of illegal drug that is being trafficked (through images of pills, mushrooms, syrups, etc.) and then fuse these image features with text features from the text vectorization technique and embed them into a deep learning model for achieving greater accuracy in detecting drug-related posts, thereby identifying the drug peddlers.

## 5. Conclusions

Contrary to popular belief, urbanization in most countries is not the result of poor regional planning, but of the tremendous benefits that a city provides to its residents. People move to cities for a variety of reasons, but one of the most important ones is the chance to learn and grow. It has been observed that our individual health and well-being are impacted by the health and well-being of the communities in which we live, work, and play, yet our health systems have frequently been organized around the needs of individuals. This was made clear by our global experience of the COVID-19 pandemic. We predict that “smart health communities”, which “reimagine public health”, “integrate well-being into urban design from the outset”, and “proactively address the drivers of health”, or the social, environmental, and economic factors that contribute to health outcomes, will become increasingly common in cities. In this paper, we found that the most common mental illnesses in cities are major depressive disorder, anxiety disorder, and substance use disorder. We have also identified the factors that contribute to their development in cities. In general, we have identified multimodal feature fusion as a technique that can help in the real-time detection and prediction of these illnesses. We have provided four use-cases to identify how multimodal feature fusion can help in the detection and prevention of common mental disorders.

## Figures and Tables

**Table 1:** Prevalence of depression in the world [19,20,21].

Rank	Most Depressed Countries	Prevalence
1	Ukraine	**6.30%**
2	United States	**5.90%**
3	Brazil	**5.80%**
4	Portugal	**5.70%**
5	Finland	**5.60%**
6	Russia	**5.50%**
7	New Zealand	**5.40%**
8	Germany	**5.20%**
9	United Arab Emirates	**5.10%**
10	Kuwait	**5.00%**

**Table 2:** Prevalence rates of different anxiety disorders.

Anxiety Disorder	Prevalence Rate (12 Months-Lifetime) Alonso et al. [31]	Prevalence Rate (12 Months-Lifetime) Wittchen et al. [32]	Prevalence Rate (12 Months-Lifetime) Kessler et al. [33]
Specific phobias	5.4–8.3	0.8–11.1	10.1–13.8
Social phobias	1.6–2.8	0.6–7.9	8.0–13.0
Panic disorder	0.7–1.6	0.7–3.1	3.1–5.2
Agoraphobia	0.3–0.8	0.1–10.5	1.7–2.6
GAD	0.9–2.8	0.2–4.3	2.9–6.2
**Overall**	**8.4–14.5**	**11.1–13.0**	**21.3–33.7**

**Table 3:** Prevalence of various substances by region in the year 2019.

Region	Prevalence (%) of Cannabis	Prevalence (%) of Opioids	Prevalence (%) of Opiates	Prevalence (%) of Cocaine	Prevalence (%) of Amphetamines	Prevalence (%) of Ecstasy
Africa	8.5	1.66	1.08	0.58	0.82	1.13
Americas	9.23	2.04	0.49	1.70	1.41	0.56
Asia	3.11	1.56	0.97	0.08	0.44	0.59
Europe	5.82	0.70	0.60	1.02	0.56	0.85
Oceania	12.42	2.76	0.12	2.73	1.33	2.22

**Table 4:** Quantifying stress/anxiety and threat levels under cyber attacks in smart cities.

Crime Committed by	Stress/Anxiety Measure on Scale 1 (Low) to 4 (High)	Threat Perception Measure on Scale 1 (Low) to 4 (High)
No terrorism group	2.3	2.9
Non-lethal cyberterrorism group (data, money, asset loss, etc.)	3.5	3.4
Lethal cyberterrorism group (deaths and injuries)	3.6	3.6
Lethal conventional terrorism group (deaths and injuries)	4.0	3.8

## Data Availability

Not Applicable.
